# Heart murmurs in the general population: diagnostic value and prevalence from the Tromsø Study

**DOI:** 10.1136/heartjnl-2024-325499

**Published:** 2025-08-01

**Authors:** Anne Herefoss Davidsen, Stian Andersen, Peder Andreas Halvorsen, Juan Carlos Aviles Solis, Henrik Schirmer, Hasse Melbye

**Affiliations:** 1 Department of Community Medicine, UiT The Arctic University of Norway, Tromsø, Norway; 2 Institute of Clinical Medicine, Campus Ahus, University of Oslo Faculty of Medicine, Oslo, Norway; 3 Department of Cardiology, Akershus University Hospital, Lorenskog, Norway

**Keywords:** Valvular Heart Disease, Heart Valve Diseases, Clinical Competence

## Abstract

**Background:**

Heart auscultation is a widely used and cost-effective clinical tool for detecting valvular heart disease (VHD), particularly in primary care. However, existing evidence on its diagnostic accuracy is limited by small sample sizes, specialist-led studies and high-prevalence settings. Robust population-based data are lacking. This study aimed to assess the prevalence and diagnostic accuracy of heart murmurs for identifying VHD in a general adult population, using echocardiography as the reference standard.

**Methods:**

We conducted a diagnostic accuracy study within the Seventh Tromsø Study (2015–2016), involving 2082 participants aged ≥40 years (mean age 63 years). Heart sounds were recorded at four chest locations and independently classified by general practitioners (GPs) blinded to echocardiographic results. Murmurs were graded and categorised as systolic or diastolic. Clinically significant VHD was defined as ≥mild aortic stenosis (AS) or ≥moderate aortic regurgitation (AR) or mitral regurgitation (MR). We calculated sensitivity, specificity, predictive values and likelihood ratios for murmur detection.

**Results:**

GPs detected heart murmurs in 487 participants (23%). Significant VHD was identified in 392 participants (19%), but only 139 of them (35.5%) had an audible murmur. Systolic murmurs detected all cases of AS (sensitivity 100%, specificity 78%). Sensitivity was lower for AR (43%) and MR (29%), while specificity of distinct murmurs exceeded 94% across all VHD types. Diastolic murmurs were rare (n=9) but highly specific (>99%). Among participants with murmurs, age ≥70 years (OR 2.0, 95% CI 1.2 to 3.4), male sex (OR 3.3, 95% CI 2.0 to 5.3) and previous myocardial infarction (OR 2.3, 95% CI 1.0 to 5.2) were independently associated with VHD.

**Conclusion:**

In this general adult population, heart auscultation by GPs identified murmurs in nearly one in four individuals and showed high specificity but limited sensitivity for diagnosing VHD. Auscultation remains a valuable initial screening tool—especially for AS—but should be complemented by echocardiography in older or high-risk individuals.

**WHAT IS ALREADY KNOWN ON THIS TOPIC**

- Most studies on heart auscultation are hospital based, involve small or selected populations and use specialist auscultators.

**WHAT THIS STUDY ADDS**

- This large, population-based study shows that general practitioners detect murmurs in nearly one in four adults, but most do not reflect clinically significant valvular disease. Sensitivity was low for most valvular heart diseases except aortic stenosis.

**HOW THIS STUDY MIGHT AFFECT RESEARCH, PRACTICE OR POLICY**

- Auscultation remains useful—particularly for identifying aortic stenosis—but should be followed by echocardiography in selected high-risk individuals. The findings support more targeted use of diagnostic imaging and greater awareness that many murmurs in general practice are benign.

## Introduction

 Heart sounds have been used for centuries to evaluate different heart conditions. The invention of the stethoscope in 1816 revolutionised heart auscultation by enabling physicians to distinguish between subtle differences in heart sounds and detect murmurs.^1 2^ Valvular heart disease (VHD), which includes stenosis and regurgitation of the valves of the heart, causes heart murmurs by creating turbulent blood flow across the abnormal heart valves.^2^ The prevalence of VHD is growing worldwide due to improved survival after heart disease and an ageing population.^3^ A population-based study from England found a prevalence of VHD of 11.3% among those ≥65 years and estimated that the prevalence will double before 2050.^4^ Echocardiography is the current reference standard for assessing VHD.^5^ However, the stethoscope is widely available and cheap, and the examination is quickly done. Hence, heart auscultation is still the most common examination when VHD is suspected in primary care.

Previous studies of the diagnostic accuracy of murmur detection in VHD have shown considerable variation in sensitivity and specificity (30–100% and 28–100%, respectively).^6^ Most studies are relatively small and from hospital settings, whereas evidence from low prevalence settings is scarce. Two studies done in a primary care setting both included patients ≥65 years without symptoms of VHD. One (n=167) found that the sensitivity of cardiac auscultation in aortic stenosis (AS) was 88%.^7^ However, the other study (n=251) found a sensitivity of only 44% in finding significant VHD, when two experienced general practitioners (GPs) auscultated.^8^ Moderate inter-rater agreement on heart murmurs adds to the limitation of cardiac auscultation.^9^ To our knowledge, however, no large-scale studies have been done in a low-prevalence population with primary care physicians as auscultators.^6^

Our primary aims were to perform a large-scale study of the prevalence of murmurs in an adult population, and to assess the diagnostic properties of heart sound characteristics with respect to VHD in a low-prevalence population. A secondary aim was to explore potential predictors of VHD, such as comorbidities and other characteristics, among individuals with murmurs.

## Methods

### Population

Inhabitants of Tromsø municipality, Northern Norway, aged 40 years or more (n=32 591), were invited to the Seventh Tromsø Study (Tromsø7) (2015–2016). The attendance rate of the first visit was 65% (n=21 083), and 9235 were invited to a second visit, in which 90.2% attended (n=8346). The population and more details on study design and data collection are well described in an article by Hopstock *et al.*^10^

### Sample

Heart sound recordings and echocardiography were gathered in a subsample of the group invited to the second visit. The participants invited to the second visit were predefined through random selection before the first visit, consisting of 20% from the 40–59 age group and 50% from the 60–84 age group. From this group, a random sample was chosen for both heart examinations and heart sound recordings, in addition to a cohort from Tromsø6 who had undergone echocardiography in that study.^11^

### Recording of heart sounds

**Figure 1:**
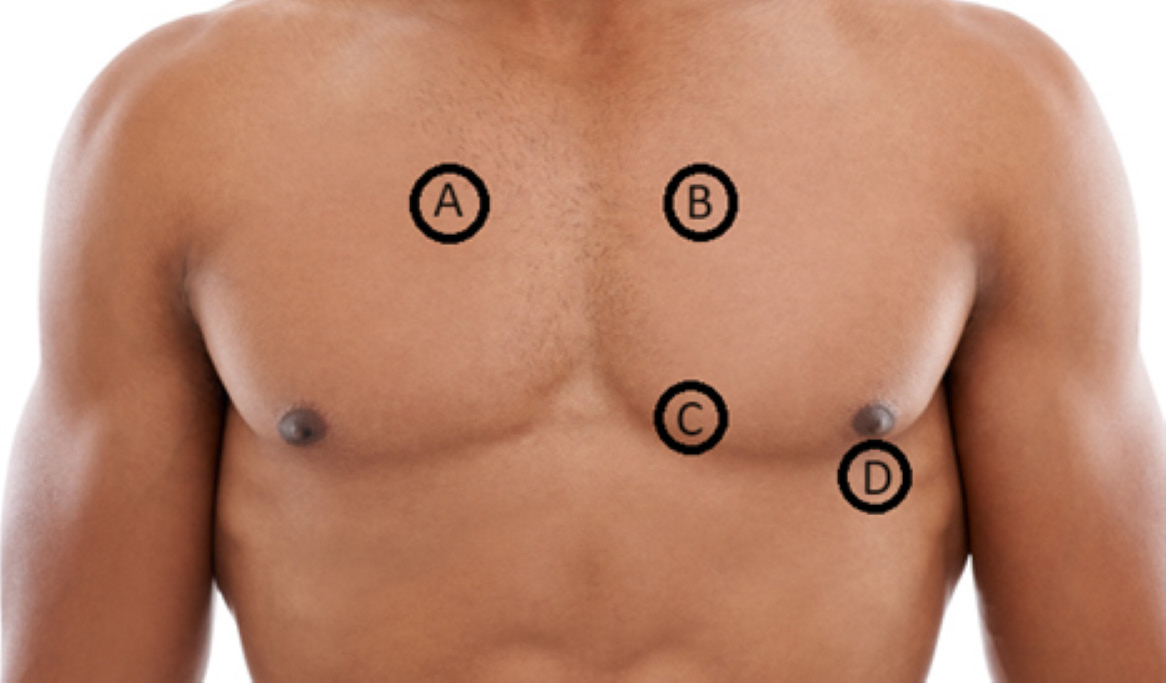
Heart sound recording sites in Seventh Tromsø Study (Tromsø7). (A) Intersection of the second intercostal space and right parasternal line. (B) Intersection of the second intercostal space and left parasternal line. (C) Intersection of the fourth intercostal space and left parasternal line. (D) Intersection of the fifth intercostal space and mid-clavicular line.

Heart sounds were recorded at four sites on the chest (figure 1).

The recordings were captured using a modified analogue stethoscope (Littmann Classic II, 3M, Maplewood, Minnesota, USA) with the tube cut 10 cm from the chest piece. A microphone (MKE 2-EW, Sennheiser Electronic, Wedemark, Germany) was inserted in the chest piece-end of the tube and wirelessly connected to a microphone system (EW 112-P G3-G, Sennheiser Electronic). Recording was controlled by a laptop using a wireless control (R700, Logitech Europe, Lausanne, Switzerland), capturing the sound via an external sound card (Scarlett 2i2, Focusrite Audio Engineering, High Wycombe, UK) and labelled by personalised software. All recordings were 10 s long and saved as ‘.wav’ format. Adobe Audition CS6 software (Adobe, San Jose, California, USA) was used for audio and spectrogram visualisation and Microsoft Access for creating the reference database. Spectrograms were not used systematically for classification purposes but could sometimes be used to assist in visually identifying the beginning of systole. In some cases, the murmur was evident in the spectrogram, and when in doubt, the spectrogram was used as visual aid in the consensus discussion (examples of spectrograms in online supplemental file 2).

We used a three-step process to create a reference classification that included three GPs (SA, AHD, HM) and a cardiologist (HS), and all were blinded to the echocardiography results and other tests reported in Tromsø7. The process has been described in another article,^9^ but will be summarised briefly here. First, four physicians (the three GPs and the cardiologist) separately classified the first 400 recordings as either normal, systolic murmur, diastolic murmur or ‘noise’, and afterwards any disagreement was resolved by consensus discussions. Second, two GPs (AHD and SA) listened to the remaining recordings and classified the heart sounds in the same manner. Disagreements were resolved in a consensus meeting with a third GP (HM). If there still was disagreement, a cardiologist (HS) was asked for a final decision. A participant was classified as having a murmur if there was consensus that we heard a murmur at one or more of the auscultation sites. If a murmur was annotated, we graded the murmur on a scale from 1 to 6. We also registered whether the second heart tone was audible.

### Echocardiography

The echocardiography examinations were performed with a GE Vivid E9 (GE Medical, Horten, Norway) ultrasound scanner, the same day as the heart sound recording. An experienced ultrasound technician performed the examination, and the assessment of the results was done by an experienced physician, supervised by coauthor HS. The severity of AS and mitral stenosis (MS) was graded on a scale from 0 to 3 (absent, mild, moderate and severe), and the severity of aortic regurgitation (AR) and mitral regurgitation (MR) on a scale from 0 to 4 (absent, trace, mild, moderate and severe). The severity grading was done in accordance with the European Society of Cardiology and the European Association for Cardio-Thoracic Surgery guidelines.^12^ In the analyses, we included mild, moderate and severe AS, as well as moderate and severe AR and MR as ‘significant VHDs’, that is, VHDs in need of follow-up according to the guidelines.

### Data analysis

We calculated prevalences of murmur and different VHDs. Frequencies were compared using χ^2^ statistics. Analysis of variance was used to compare means. We calculated sensitivity and specificity of murmurs and absent second heart tone with respect to VHD using echocardiography as gold standard. We used logistic regression to explore potential predictors for VHD. We included the following independent variables: breathlessness when walking fast or uphill, chest pain when walking fast or uphill, age, sex, systolic blood pressure, body mass index (BMI, ≥30), self-perceived health, previous heart attack, stroke or hypertension, atrial fibrillation (in ECG), left ventricle hypertrophy (in ECG), haemoglobin level (below normal, men and women) and smoking status.

We used IBM SPSS Statistics V.28.0.0.0 (190) for the analyses.

### Patient and public involvement

This population-based study used data from Tromsø7, and patients or the public were not involved in the design of the study.

## Results

**Figure 2:**
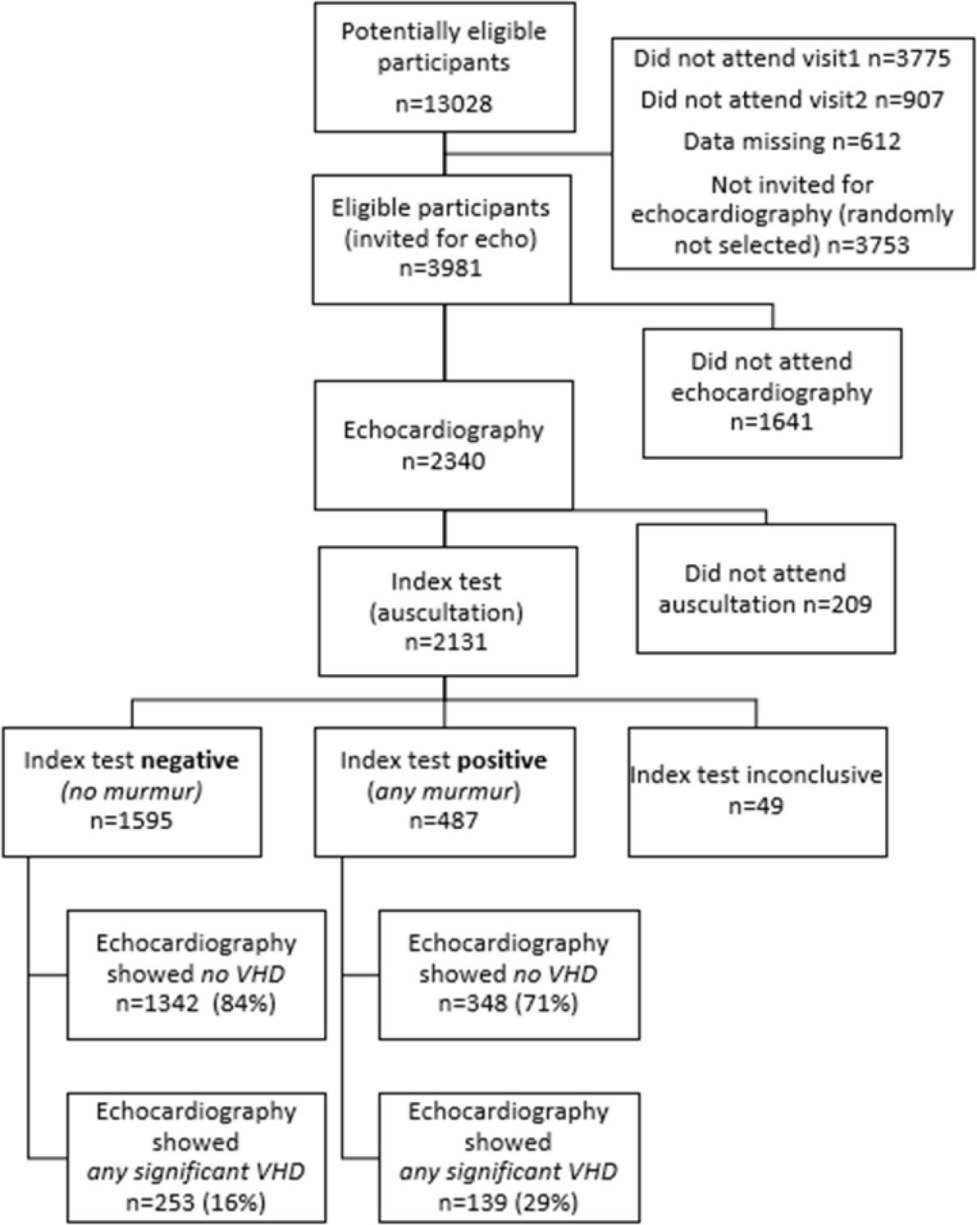
Standards for Reporting Diagnostic Accuracy (STARD) flow chart for auscultation (index test) to diagnose significant valvular heart disease (VHD) (significant=mild to severe aortic stenosis, or moderate to severe mitral regurgitation or aortic regurgitation). The reference standard was echocardiography.

We recorded heart sounds from 2409 participants and echocardiography from 2340 participants. A total of 2131 participants had both echocardiography and heart sound recordings. Among those, 49 could not be classified due to noise. Thus, we ended up with 2082 classifiable participants (figure 2).

The demographics are presented in table 1. Mean age was 64 years, 54% were female, 12% were smokers, 67% had a BMI >25, 37% reported one or more comorbidities and 68% considered their own health ‘good’ or ‘excellent’. Non-participants were defined as those attending the second visit, but without echocardiography (not invited or did not attend), and they had similar characteristics (table 1).

**Table 1:** Participant characteristics

	Total participants with echocardiography n*	Any murmur n (%)	No murmur n (%)	P value†	Tromsø7 participants without‡ echocardiography
All	2082	487 (23.4)	1595 (76.6)		5394
Sex					
Men	949 (45.6)	169 (34.7)	780 (48.9)		2429 (45)
Women	1133 (54.4)	318 (65.3)	815 (51.1)	<0.001	2965 (55)
Age					
40–49	272 (13.1)	31 (6.4)	241 (15.1)		768 (14.2)
50–59	362 (17.4)	50 (10.3)	312 (19.6)		913 (16.9)
60–69	755 (36.2)	141 (29.0)	614 (38.5)		2252 (41.8)
70+	693 (33.3)	265 (54.4)	428 (26.8)	<0.001§	1461 (27.1)¶
Mean	64.0	68.8	62.5	<0.001**	62.8
Smoking					
Current	246 (11.8)	42 (8.7)	204 (12.9)		634 (11.9)
Previous	1008 (48.4)	233 (48.4)	775 (49.1)		2545 (47.8)
Never	805 (38.7)	206 (42.8)	599 (38.0)	0.02	2140 (40.2)
BMI					
<20	57 (2.7)	13 (2.7)	44 (2.8)		115 (2.1)
20–24.9	635 (30.5)	151 (31.1)	484 (30.4)		1567 (29.1)
25–29.9	924 (44.4)	206 (42.4)	718 (45.0)		2423 (44.9)
≥30	464 (22.3)	116 (23.9)	348 (21.8)	0.72§	1270 (23.5)
Mean	27.2	27.2	27.1		27.4
Self-reported disease					
Hypertension	756 (36.3)	249 (52.8)	507 (32.5)	<0.001	1822 (34.8)
Myocardial infarction	120 (5.8)	41 (9.0)	79 (5.0)	0.002	228 (4.4)¶
Atrial fibrillation	175 (8.4)	43 (9.5)	132 (8.3)	0.56	394 (7.6)
Heart failure	71 (3.4)	27 (6.0)	44 (2.8)	0.002	128 (2.5)¶
Cerebral stroke	73 (3.5)	30 (6.5)	43 (2.8)	<0.001	167 (3.2)
Diabetes	134 (6.4)	44 (9.4)	90 (5.6)	0.005	340 (6.5)
COPD	85 (4.1)	20 (4.3)	65 (4.2)	0.94	221 (4.3)
Asthma	240 (11.5)	50 (10.8)	190 (12.2)	0.40	587 (11.3)
Self-perceived health					
Very bad or bad	94 (4.5)	22 (4.6)	72 (4.5)		261 (4.9)
Neither good nor bad	546 (26.2)	156 (32.4)	390 (24.7)		1495 (28.0)
Good	1140 (54.8)	250 (52.0)	890 (56.4)		2892 (54.2)
Excellent	278 (13.4)	53 (11.0)	225 (14.3)	0.006§	685 (12.8)
Hgb					
Mean Hgb, men	14.9	14.6	15.0		14.9
% below normal††	4.8	10.1	3.7		3.9
Mean Hgb, women	13.6	13.6	13.6		13.6
% below normal††	3.2	3.5	3.1		1.7

* Some values missing because of missing answers from questionnaire.

† Pearson χ2 for difference between those who have a murmur and those who do not have a murmur.

‡ Participated on day 2 of Tromsø7, but was either not invited for echocardiography (n=3753) or did not attend (n=1641)*.*

§ Pearson χ2 trend*.*

¶ P value <0.05 for difference between participants with echocardiography and participants without echocardiography*.*

** T-test*.*

†† Ref Hgb: https://labhandbok.unn.no/medisinsk-biokjemi/hemoglobin-article1958-816.html

BMI, body mass index; COPD, chronic obstructive pulmonary disease; Hgb, haemoglobin; Tromsø7, Seventh Tromsø Study.

### Prevalences

The prevalence of any murmur was 487 (23%). Among those, 484 had a systolic murmur (31% distinct) and nine had a diastolic murmur. Murmurs were significantly more common in women, among participants ≥70 years and in those with hypertension, diabetes, history of myocardial infarction and heart failure (table 1). A murmur was more often heard in the right second intercostal space.

A total of 392 (19%) participants had a significant VHD. Of these, 45 participants had a mild to severe AS (34 were aged 70 years and above), 286 had a moderate to severe MR and 148 had a moderate to severe AR. Only three participants had a clinically significant MS (moderate to severe), and all of these were combined with other VHDs. Analysis has therefore not been done specifically on MS. A total of 79 participants had more than one significant VHD. Significant VHD was more common among men than in women (21.5% vs 16.6%, χ^2^=8.12, p=0.004).

**Figure 3:**
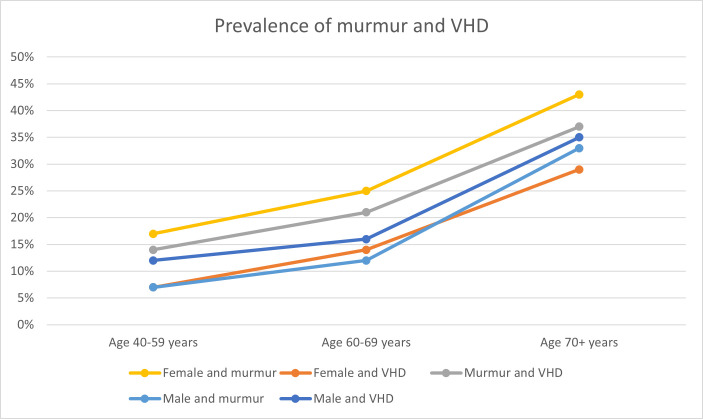
Prevalence of murmur and valvular heart disease (VHD) related to age and sex, and the combination of murmur and VHD related to age in the Seventh Tromsø Study (Tromsø7).

The prevalence of both murmur and VHD increased with age (figure 3).

### Correlation of VHD and murmur

**Figure 4:**
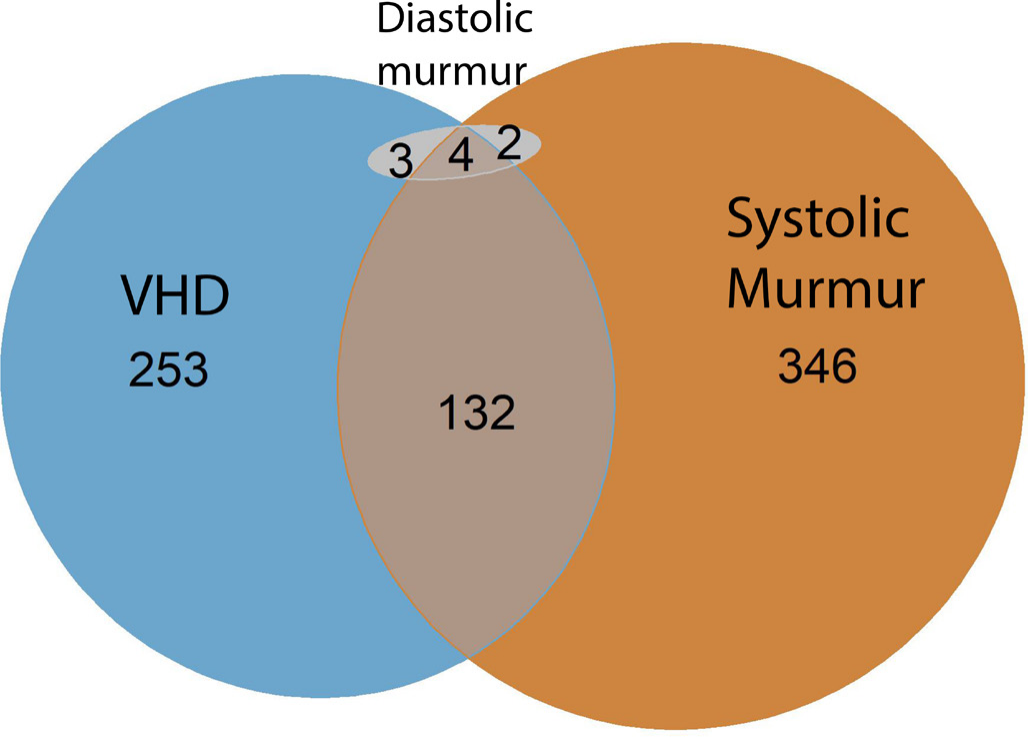
Euler diagram of the correlation between VHD and murmur in Tromsø7. Significant VHD=mild to severe AS, moderate to severe AR or MR. AR, aortic regurgitation; AS, aortic stenosis; MR, mitral regurgitation; Tromsø7, Seventh Tromsø Study; VHD, valvular heart disease.

Among participants with a significant VHD, only a minority (35.5%) had an audible murmur. A notable exception was AS, where all had an audible murmur. In AR, systolic murmurs were more common than diastolic murmurs (online supplemental e-table 1). Only nine participants had a diastolic murmur, of whom all had AR, six had a moderate or severe AR and the last three had a mild AR (online supplemental e-table 1). The correlation of VHD and murmur is illustrated in figures4  5.

**Figure 5:**
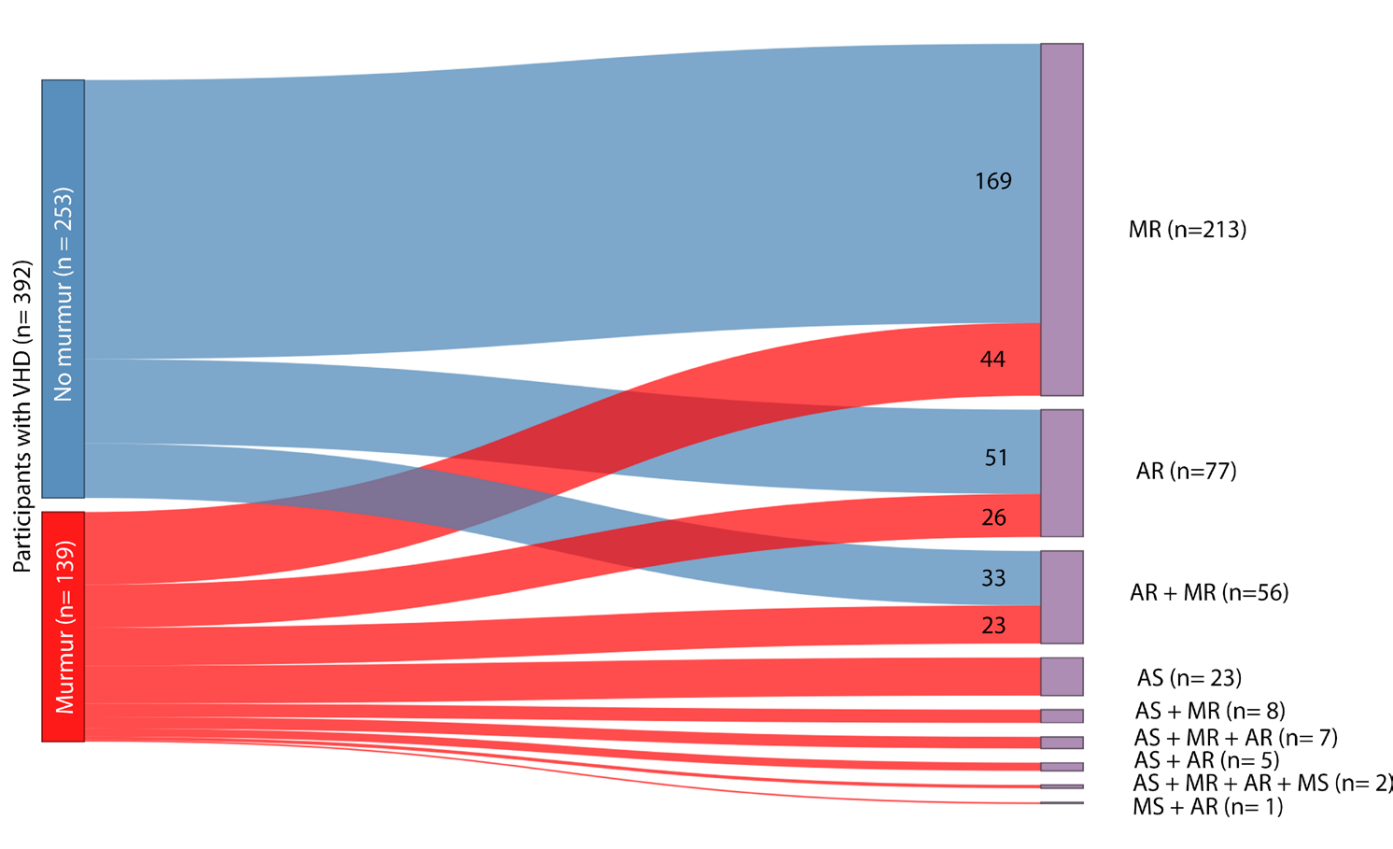
Participants with VHD and its relationship to presence/absence of murmur. Significant VHD=mild to severe AS, moderate to severe AR or MR. AR, aortic regurgitation; AS, aortic stenosis; MR, mitral regurgitation; MS, mitral stenosis; VHD, valvular heart disease.

Indices of diagnostic accuracy of murmurs with respect to murmur grade and different VHDs are presented in detail in table 2 (confusion matrices in online supplemental file 3). All cases of a specific VHD were analysed regardless of the presence of other VHDs. The prevalence of the different VHDs, including the different combinations of VHDs, is presented in online supplemental e-table 1. Sensitivity of systolic murmurs was generally low, except for AS, whereas the specificity of a distinct systolic murmur was 94–95%. The presence of a diastolic murmur was highly indicative of VHD with a specificity >99%, but the sensitivity was very low, even for AR. The positive predictive value (PPV) of any murmur for identifying any significant VHD was 28.5 (table 2), but this varied with age (online supplemental e-table 2). Likelihood ratios (LR) were less than 5 except for a distinct systolic murmur in the diagnosis of AS (LR 16.3, 95% CI 12.7 to 21.0) and for diastolic murmurs with respect to AR (LR 26.1, 95% CI 6.6 to 103.5).

**Table 2:** Sensitivity, specificity, positive predictive value (PPV), negative predictive value (NPV) and likelihood ratio (LR+) of murmur finding in relation to presence of significant VHD (mild to severe AS, moderate to severe AR or MR)

		Any VHD* (n=392)	AS (n=45)	AR (n=148)	MR (n=286)
Any murmur (n=487)	Sensitivity (95% CI)	35.5 (30.7 to 40.4)	100 (92 to 100)	43.2 (35.1 to 51.6)	29.4 (24.2 to 35.0)
	Specificity (95% CI)	79.4 (77.4 to 81.3)	78.3 (76.5–80.1)	78.1 (76.2 to 80.0)	77.6 (75.6 to 79.5)
	PPV (95% CI)	28.5 (25.3 to 32.0)	9.2 (8.6 to 10.0)	13.1 (11.0 to 15.6)	17.3 (14.6 to 20.3)
	NPV (95% CI)	84.1 (83.1 to 85.1)	100 (99.8 to 100)	94.7 (94.0 to 95.4)	87.3 (86.4 to 88.2)
	LR+ (95% CI)	1.7 (1.5 to 2.0)	4.6 (4.2 to 5.0)	2.0 (1.6 to 2.4)	1.3 (1.1 to 1.6)
Distinct systolic murmur (n=148)	Sensitivity (95% CI)	13.3 (10.1 to 17.0)	77.8 (62.9 to 88.8)	14.9 (9.6 to 21.6)	8.0 (5.2 to 11.8)
	Specificity (95% CI)	95.3 (94.1 to 96.2)	95.2 (94.2 to 96.1)	94.3 (93.2 to 95.3)	93.9 (92.7 to 95.0)
	PPV (95% CI)	39.4 (31.8 to 47.5)	26.5 (22.0 to 31.6)	16.7 (11.6 to 23.5)	17.4 (12.1 to 24.5)
	NPV (95% CI)	82.6 (82.0 to 83.1)	99.5 (99.1 to 99.7)	93.5 (93.1 to 93.9)	86.5 (86.1 to 86.9)
	LR+ (95% CI)	2.8 (2.0 to 3.9)	16.3 (12.7 to 21.0)	2.6 (1.7 to 4.0)	1.3 (0.86 to 2.0)
Diastolic murmur (n=9)	Sensitivity (95% CI)	1.8 (0.7 to 3.6)	0 (0 to 7.9)	4.0 (1.5 to 8.6)	1.4 (0.4 to 3.5)
	Specificity (95% CI)	99.9 (99.6 to 100)	99.6 (99.2 to 99.8)	99.8 (99.6 to 100)	99.7 (99.4 to 99.9)
	PPV (95% CI)	77.8 (42.2 to 94.4)	0	66.7 (33.6 to 88.8)	44.4 (17.8 to 74.8)
	NPV (95% CI)	81.4 (81.2 to 81.6)	97.83 (97.82 to 97.84)	93.0 (91.9 to 94.1)	86.4 (86.2 to 86.6)
	LR+ (95% CI)	15.1 (3.15 to 72.4)	0	26.1 (6.6 to 103.5)	5.0 (1.36 to 18.6)

The Seventh Tromsø Study (Tromsø7).

Significant VHD=mild to severe AS, moderate to severe AR or MR.

Distinct murmur: grade ≥3/6*.*

* Participants with one or more significant VHDs.

AR, aortic regurgitation; AS, aortic stenosis; MR, mitral regurgitation; VHD, valvular heart disease.

**Figure 6:**
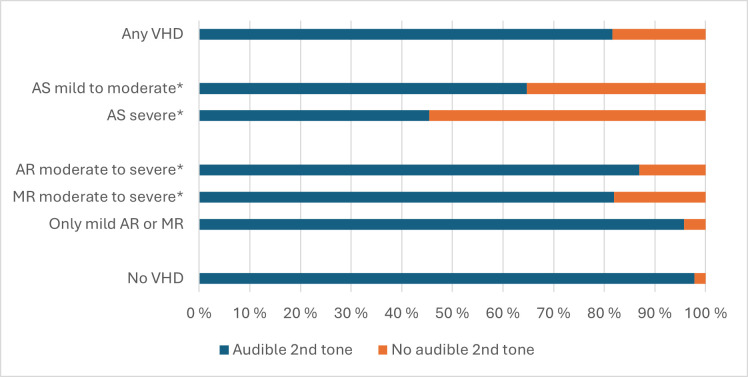
Audibility of the second heart tone among those with a VHD and a systolic murmur. Significant VHD=mild to severe AS, moderate to severe AR or MR. *Might have other VHDs in addition to the presented VHD. AR, aortic regurgitation; AS, aortic stenosis; MR, mitral regurgitation; VHD, valvular heart disease.

Among those with a systolic murmur, 7.2% had an inaudible second heart tone. This finding was highly related to severe AS (figure 6), but not sensitive enough to rule it out (sensitivity 55%, 95% CI 23% to 83%; specificity 99%, 95% CI 98.6% to 99.5%; LR 62, 95% CI 30 to 125).

In a multivariable logistic regression analysis including all participants with a murmur (n=487), we found that male sex (OR 3.3, 95% CI 2.0 to 5.3), age 70+ (OR 2.0, 95% CI 1.2 to 3.4) and a previous heart attack (OR 2.3, 95% CI 1.04 to 5.2) were associated with VHD. To illustrate, for a female younger than 70 years without a previous heart attack, the PPV of a murmur indicating a VHD is only 10%, whereas for a man ≥70 years with a history of heart attack, the PPV is 67%. However, the regression model explained less than 20% of the variance.

## Discussion

In this population-based survey, almost one in four had a murmur, and one in five had one or more significant VHDs. Murmurs were more common among women (28.1% vs 17.8%) and among those ≥70 years (38% vs 16%). In general, the sensitivity of a systolic murmur to detect VHD was only 35.5%, but for AS, it was 100%. Among those with a murmur, 28.5% had a significant VHD. The absence of a second heart tone did not distinguish well between severe and less severe AS. High age, male sex and previous heart attack increased the likelihood of VHD among individuals with murmurs.

### Strengths and limitations

An obvious strength of this study is the sample size (>2000). To our knowledge, this is by far the largest study of heart auscultation in a general, low-prevalence population using echocardiography as gold standard for diagnosing VHD. Moreover, the rigorous procedure for classification of the sound recordings strengthens the internal validity of the study. Arguably, using GPs as raters of the recordings instead of cardiologists strengthens the external validity from a primary care point of view.

Among the limitations is that only two physicians did most of the rating of heart sounds. During the course of the study, they gained considerable experience from rating more than 8000 heart sound recordings, which doubtfully represents the average GP. Adding to that, the rigorous procedures and access to spectrograms while rating the heart sounds might have compromised the external validity for clinical practice.

Another limitation is that the classification of the heart sounds is based on recordings and not real-life auscultation. Consequently, using the entire Levine’s scale was impossible, as the higher grades require the presence of a palpable thrill. A physician auscultating a patient would also be able to position the patient and adjust the stethoscope placement to improve the quality of the auscultation.

The participation rate was 65%, and among those >70 years of age, the participation rate was only 59%. There were no significant differences between the participants and the non-participants with regard to sex, age and comorbidities (table 1), but participation bias could still have influenced the results. Often, those participating are healthier than those who stay at home, especially among the oldest. Among the younger, those not participating might be more occupied at work or with other activities.

### Comparison with previous studies

The diagnostic accuracy of auscultation in our study (71%) is similar to the accuracy in a study by Steeds *et al* which was also done in a general population and with GPs auscultating,^7^ and a little higher than the accuracy in a study by Gardezi *et al*.^8^ Our study includes considerably more participants, and the study by Steeds *et al* did not examine all patients with a full echocardiography.

The prevalence of significant VHD is comparable to previous studies, and the increase in prevalence with older age is also well known.^13^ More men than women have a significant VHD, as shown in large population-based studies previously.^14^ More recent studies suggest that the prevalence of AS is higher in men (this is the case in our study as well), whereas the prevalence of MR is higher in women (we have not seen this difference in our study).^15 16^

The use of spectrograms has been shown to improve the classification of lung sound recordings,^17^ and this could possibly be the case for heart sounds as well. The use of phonocardiograms has been shown to increase the rate of correct diagnosis in heart auscultation.^18^

### Implications for practice and research

As the stethoscope turned 200 years in 2016, many questioned whether it is still relevant.^1 18 19^ Most concluded that it is too early to bury the stethoscope, but there is a need for improvement: more training,^19^ use of electronic stethoscopes and computer programs or apps,^1 18 19^ as well as realistic expectations—that is, distinguishing normal from abnormal heart sounds rather than diagnosing specific diseases.^19^

The diagnostic properties of auscultation may seem poor at first glance, but in comparison with other frequently used tests in general practice, it performs quite well. For example, the sensitivity of C-reactive protein >100 mg/mL to diagnose community-acquired pneumonia was in one study only 59% (specificity 79%),^20^ and the sensitivity of liver function tests to diagnose advanced liver fibrosis has varied from 10% to 67%.^21^ These numbers are comparable to the diagnostic accuracy of heart auscultation. PPV for warning signs of cancer has proven considerably lower (0.8-3.8%)^22^ than the PPVs for heart auscultation in our study, but still there is a common agreement for referral of those patients for further examinations. In comparison, adults with a new murmur will have a much higher likelihood of having a significant disease, possibly in need of surgical treatment, than patients referred with a warning sign of cancer. Further research should be done on the pathophysiological mechanisms of the lack of a murmur in some patients with VHD, and the possible correlation to heart failure.

In 2018, the editor-in-chief of the international journal *Heart*, Catherine Otto, wrote, “it’s time to turn to more effective technology—ultrasound, not acoustic sound,”^23^ and Mehta *et al* conclude that ultrasound is so superior to physical examination that it makes any argument to not use it irrelevant.^24^ Narula *et al* argue not to replace auscultation, but to add point-of-care ultrasound (POCUS) to the bedside physical examination.^25^ A review from 2020 concludes that ‘POCUS can serve as a screening tool and guide the management of patients with VHD’, and finds that it can prevent unnecessary imaging.^26^ However, ultrasound is still more expensive and less accessible for most physicians, and there is a need for physicians to be well trained in ultrasound before we discard the stethoscope.^1^ Introducing ultrasound to practising physicians has proven difficult for several reasons: reluctance to obtain more training, time constraints (ultrasound is considered more time consuming than auscultation) and lack of financial incentives.^24^ Our findings show that auscultation is good for finding AS, including mild cases. This is important since AS is increasing and can often be treated with transcatheter aortic valve implantation. As the presence of VHD increases with age, finding the right cut-off for referring the elderly to further examinations might be important in the future with an increasing population of elderly people. A study from 2021 found that it was feasible for a GP to screen elderly patients for AS in a primary care setting with a handheld ultrasound device.^7^

A possible intermediate step before a more widespread implementation of ultrasound could be the use of electronic stethoscopes and computer programs or applications helping the physician to interpret the heart sounds of the patient. A recent study based on the same material as ours showed high accuracy in finding VHDs using a murmur detection algorithm with symptoms, age and gender included as predictors.^27^

And as always in medicine, no test stands alone in the diagnostic workup. Further research should focus on auscultation in clinical practice and how it should be used by the doctor in the total evaluation of a patient.

## Conclusion

Heart auscultation by GPs identified murmurs in nearly one in four individuals in this study. Auscultation had high specificity but limited sensitivity for diagnosing VHD. The only VHD for which heart auscultation was highly sensitive was AS (100%). It may be a valuable first step but should be complemented by echocardiography in high-risk individuals. Older age, male sex and previous heart attack increased the likelihood of VHD among individuals with murmurs.

## Supplementary material

10.1136/heartjnl-2024-325499 online supplemental file 1

10.1136/heartjnl-2024-325499 online supplemental file 2

10.1136/heartjnl-2024-325499 online supplemental file 3

## Data Availability

Data may be obtained from a third party and are not publicly available.
