# Supplementary material for: Heart murmurs in the general population: diagnostic value and prevalence from the Tromsø Study
Source: Heart. 2025 Aug 1;112(2):e325499. doi: 10.1136/heartjnl-2024-325499 (PMC12772608; doi:10.1136/heartjnl-2024-325499)
Supplement: online supplemental file 1 [file heartjnl-112-2-s001.docx]

E-tables

E-table 1: Murmur and valvular heart disease

| Type of VHD (significant VHD** unless otherwise noted) | Murmur | | |  |
| --- | --- | --- | --- | --- |
|  | *Systolic, faint*  *N=336*  n *(%*)* | *Systolic, distinct*  *N=148*  n *(%*)* | *Diastolic murmur*  *N=9*  n *(%*)* | *Any murmur N=487*  n *(%*)* |
| Any significant VHD**  n=392 | 73 (19) | 63 (16) | 6 (2) | 139 (35) |
| AS alone  n=23 | 5 (22) | 18 (78) | 0 | 23 (100) |
| AS combined with AR (n=5), MR (n=8), AR and MR (n=7) or AR, MR and MS (n=2) n=22 | 3 (14) | 19 (86) | 0 | (100) |
| AR alone  n=77 | 17 (22) | 7 (9) | 3 (4) | 26 (34) |
| AR combined with MR only  n=56 | 15 (27) | 7 (13) | 3 (4) | 23 (41) |
| MR alone  n=213 | 33 (15) | 11 (5) | 1 (0.5) | 44 (21) |
| Mild AR (n=72) and/or mild MR (n=473) and/or mild MS (n=11), without other VHD n=518 | 88 (17) | 33 (6) | 2 (0.4) | 121 (23) |
| No VHD (not even mild AR/MR/MS)  n=1172 | 175 (15) | 52 (4) | 0 | 227 (19) |

VHD=valvular heart disease. AS=aortic stenosis. AR= aortic regurgitation. MR=mitral regurgitation.
*%=percentage of those with the relevant VHD who have the finding.
**Significant VHD=mild to severe AS, or moderate to severe MR or AR.

E-table 2: Diagnostic usefulness of auscultation finding and valvular heart disease in relation to age

|  | No VHD | Any VHD | Sensitivity | Specificity | PPV | NPV | LR+ |
| --- | --- | --- | --- | --- | --- | --- | --- |
| Age<70y. and murmur  (n=222) | 181 | 41 | 23.8 (17.7-30.9) | 85.1 (83-87.1) | 18.5% | 88.8% | 1.6 (1.2-2.2) |
| Age≥70y. and murmur (n=265) | 167 | 98 | 44.6 (37.9-51.4) | 64.7 (60.2-69) | 37% | 71.5% | 1.3 (1.0-1.5) |

VHD=valvular heart disease. *=mild to severe aortic stenosis, moderate to severe MR, moderate to severe MS and / or moderate to severe AR. PPV=positive predictive value. NPV=negative predictive value. LR+=positive likelihood ratio. y.=years
