# Supplementary material for: Heart murmurs in the general population: diagnostic value and prevalence from the Tromsø Study
Source: Heart. 2025 Aug 1;112(2):e325499. doi: 10.1136/heartjnl-2024-325499 (PMC12772608; doi:10.1136/heartjnl-2024-325499)
Supplement: online supplemental file 2 [file heartjnl-112-2-s002.docx]

# Spectrograms

Normal heart sounds


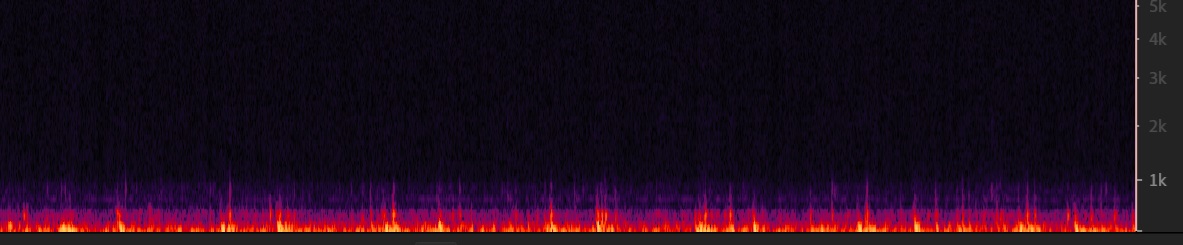


Systolic murmur


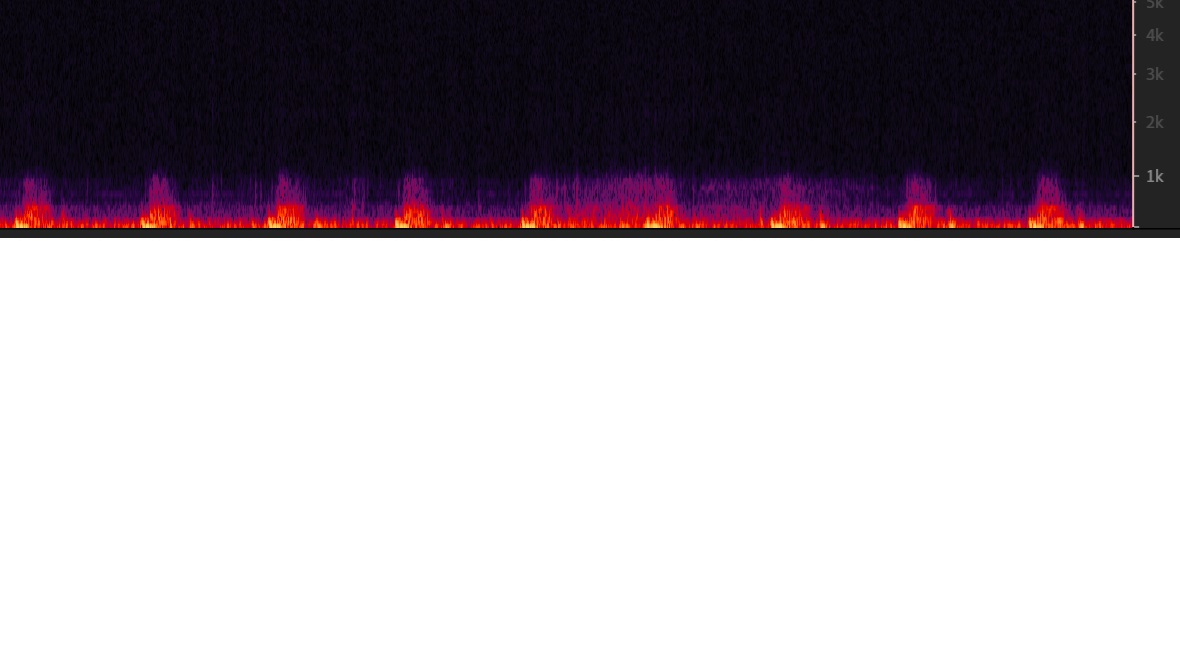


Diastolic murmur


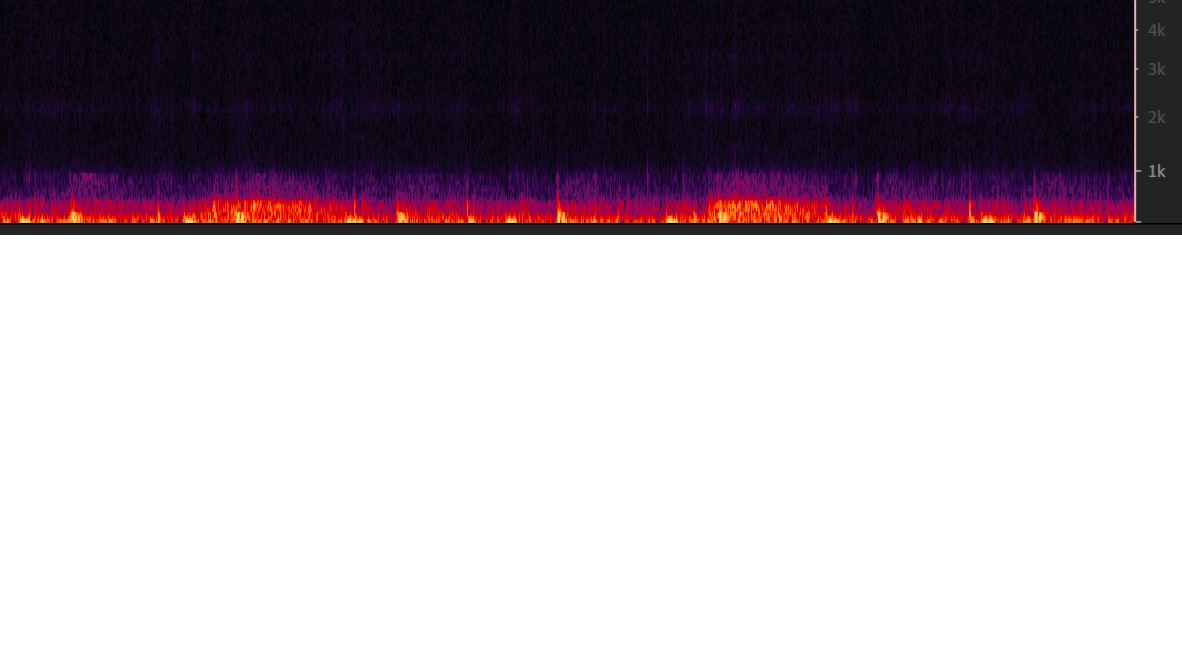


1k on vertical axis stands for 1000 Hz
