# Supplementary material for: Heart murmurs in the general population: diagnostic value and prevalence from the Tromsø Study
Source: Heart. 2025 Aug 1;112(2):e325499. doi: 10.1136/heartjnl-2024-325499 (PMC12772608; doi:10.1136/heartjnl-2024-325499)
Supplement: online supplemental file 3 [file heartjnl-112-2-s003.docx]

**Confusion matrices**

|  |  | Any significant VHD | |  |
| --- | --- | --- | --- | --- |
|  |  | + | - |  |
| Any murmur | + | 139 | 348 | 487 |
|  | - | 253 | 1342 | 1595 |
|  |  | 392 | 1690 | 2082 |

ANY MURMUR

|  |  | Aortic stenosis | |  |
| --- | --- | --- | --- | --- |
|  |  | + | - |  |
| Any murmur | + | 45 | 442 | 487 |
|  | - | 0 | 1595 | 1595 |
|  |  | 45 | 2037 | 2082 |

|  |  | Aortic regurgitation | |  |
| --- | --- | --- | --- | --- |
|  |  | + | - |  |
| Any murmur | + | 64 | 423 | 487 |
|  | - | 84 | 1511 | 1595 |
|  |  | 148 | 1934 | 2082 |

|  |  | Mitral regurgitation | |  |
| --- | --- | --- | --- | --- |
|  |  | + | - |  |
| Any murmur | + | 84 | 403 | 487 |
|  | - | 202 | 1393 | 1595 |
|  |  | 286 | 1796 | 2082 |

DISTINCT SYSTOLIC MURMUR

|  |  | Any significant VHD | |  |
| --- | --- | --- | --- | --- |
|  |  | + | - |  |
| Distinct systolic murmur | + | 63 | 85 | 148 |
|  | - | 329 | 1605 | 1934 |
|  |  | 392 | 1690 | 2082 |

|  |  | Aortic stenosis | |  |
| --- | --- | --- | --- | --- |
|  |  | + | - |  |
| Distinct systolic murmur | + | 37 | 111 | 148 |
|  | - | 8 | 1926 | 1934 |
|  |  | 45 | 2037 | 2082 |

|  |  | Aortic regurgitation | |  |
| --- | --- | --- | --- | --- |
|  |  | + | - |  |
| Distinct systolic murmur | + | 26 | 122 | 148 |
|  | - | 122 | 1812 | 1934 |
|  |  | 148 | 1934 | 2082 |

|  |  | Mitral regurgitation | |  |
| --- | --- | --- | --- | --- |
|  |  | + | - |  |
| Distinct systolic murmur | + | 33 | 115 | 148 |
|  | - | 253 | 1681 | 1934 |
|  |  | 286 | 1796 | 2082 |

DIASTOLIC MURMUR

|  |  | Any significant VHD | |  |
| --- | --- | --- | --- | --- |
|  |  | + | - |  |
| Diastolic murmur | + | 7 | 2 | 9 |
|  | - | 256 | 1342 | 1598 |
|  |  | 392 | 1690 | 2082 |

|  |  | Aortic stenosis | |  |
| --- | --- | --- | --- | --- |
|  |  | + | - |  |
| Diastolic murmur | + | 0 | 9 | 9 |
|  | - | 45 | 2028 | 2073 |
|  |  | 45 | 2037 | 2082 |

|  |  | Aortic regurgitation | |  |
| --- | --- | --- | --- | --- |
|  |  | + | - |  |
| Diastolic murmur | + | 6 | 3 | 9 |
|  | - | 142 | 1931 | 2073 |
|  |  | 148 | 1934 | 2082 |

|  |  | Mitral regurgitation | |  |
| --- | --- | --- | --- | --- |
|  |  | + | - |  |
| Diastolic murmur | + | 4 | 5 | 9 |
|  | - | 282 | 1791 | 2073 |
|  |  | 286 | 1796 | 2082 |

INAUDIBLE SECOND HEART TONE

|  |  | Aortic stenosis | |  |
| --- | --- | --- | --- | --- |
|  |  | + | - |  |
| 2nd heart tone | -not audible | 18 | 17 | 35 |
|  | -audible | 27 | 422 | 449 |
|  |  | 45 | 439 | 484 |
